# Gene Expression Profile of the Hippocampus of Rats Subjected to Chronic Immobilization Stress

**DOI:** 10.1371/journal.pone.0057621

**Published:** 2013-03-27

**Authors:** Xiao-Hong Li, Jia-Xu Chen, Guang-Xin Yue, Yue-Yun Liu, Xin Zhao, Xiao-Ling Guo, Qun Liu, You-Ming Jiang, Ming-Hua Bai

**Affiliations:** 1 School of Pre-clinical Medicine, Beijing University of Chinese Medicine, Beijing, China; 2 School of Pre-clinical Medicine, Guangxi University of Chinese Medicine, Nanning, China; 3 Institute of Basic Theory of TCM, China Academy of Chinese Medical Science, Beijing, China; Rutgers University, United States of America

## Abstract

**Objective:**

This study systematically investigated the effect of chronic stress on the hippocampus and its damage mechanism at the whole genome level.

**Methods:**

The rat whole genome expression chips (Illumina) were used to detect gene expression differences in the hippocampus of rats subjected to chronic immobilization stress (daily immobilization stress for 3 h, for 7 or 21 days). The hippocampus gene expression profile was studied through gene ontology and signal pathway analyses using bioinformatics. A differentially expressed transcription regulation network was also established. Real-time quantitative polymerase chain reaction (RT-PCR) was used to verify the microarray results and determine expression of the *Gabra1*, *Fadd*, *Crhr2*, and *Cdk6* genes in the hippocampal tissues.

**Results:**

Compared to the control group, 602 differentially expressed genes were detected in the hippocampus of rats subjected to stress for 7 days, while 566 differentially expressed genes were expressed in the animals experiencing stress for 21 days. The stress significantly inhibited the primary immune system functions of the hippocampus in animals subjected to stress for both 7 and 21 days. Immobilization activated the extracellular matrix receptor interaction pathway after 7 day exposure to stress and the cytokine-cytokine receptor interaction pathway. The enhanced collagen synthesis capacity of the hippocampal tissue was the core molecular event of the stress regulation network in the 7-day group, while the inhibition of hippocampal cell growth was the core molecular event in the 21-day group. For the *Gabra1*, *Fadd*, *Crhr2*, and *Cdk6* genes, RT-PCR results were nearly in line with gene chip assay results.

**Conclusion:**

During the 7-day and 21-day stress processes, the combined action of polygenic, multilevel, and multi-signal pathways leads to the disorder of the immunologic functions of the hippocampus, hippocampal apoptosis, and proliferation disequilibrium.

## Introduction

Stress response is characterized by the activation of the hypothalamus-pituitary-adrenal (HPA) axis and the subsequent increase in glucocorticoid (GC) secretion. HPA axis activation is an important adaptive and protective response to stress. However, in the chronic stress process, the HPA axis is usually in a continuous high-response state, leading to increased GC and functional disorders of the nervous, endocrine, and immune systems, among others. Previous studies suggested that a stress reaction due to high concentrations of GC is one of the main reasons for harming the body [1]–[2]. The hippocampus is the key brain region related to learning, memory, cognition, and emotion. It is also one of the most important encephalic regions that mediate stress reaction. The hippocampus has the highest content of glucocorticoid receptors (GR) in the central nervous system. Thus, during stress, high level of GC result in decreased hippocampal nerve cell plasticity, hippocampal apoptosis, and regeneration disequilibrium, thereby leading to nerve cell atrophy and loss, and eventually causing local structural and functional damage [3], [4]. Several studies have reported on the mechanism by which stress affects the functions of the hippocampus at the single gene level. However, investigations on the mechanism by which stress affects the function of the hippocampus at the whole genome level are lacking.

We have previously applied high-performance liquid chromatography, enzyme-linked immunosorbent assay, immunohistochemistry, reverse transcriptase-polymerase chain reaction (RT-PCR), and western blot assay to study the multiple indicators of the central nervous system in rat of chronic immobilization stress. We showed that chronic immobilization stress causes HPA axis disorder in rats [5]. Hippocampal GRs are increased in the early (animals subject to immobilization for 7 days) and reduced in the late (21 days) stages of chronic stress [6]. Redistribution of monoamine transmitter norepinephrine, serotonin, dopamine, 5-hydroxyindoleacetic acid, and homovanillic acid was found in the hypothalamus and hippocampus of stressed rats [7]. In the hippocampus, the protein expression of brain-derived neurotrophic factor and neurotrophin 3 was decreased, whereas that of tyrosine kinase B was up-regulated [8]. In rats, chronic stress increases the level of hypothalamus β-endorphin [9]. It also changes the mRNA expressions of corticotrophin-regulating factors 1 and 2, as well as proopiomelanocortin (POMC-1 and POMC-2) in the cerebral cortex, hypothalamus, pituitary gland, and hippocampus [10], [11].

The mechanism by which chronic stress affects the functions of the hippocampus remains unclear. In the present study, the whole genome expression chip, Illumina Ref-12 Rat, was used to investigate the differential gene expression profile of the hippocampal tissues of rats subjected to chronic immobilization stress. This chip contains 22,517 probe sequences, 22,226 of which were obtained from the database of NCBI Ref and UniGene. The changes in the gene expressions of rats and the mechanism by which chronic stress affects the function of the hippocampus were studied at the whole genome level.

## Materials and Methods

### Animals and grouping

A total of 69 male Sprague-Dawley rats (SPF grade) weighing 225 g±10 g were purchased from the Beijing Vitalriver Laboratory Animal Research Center (Animal license No.: SCXK (Beijing) 2006-0009). After adaptive feeding for one week, the rats were randomly divided into three groups of 23: control group, 7-day stress group, and 21-day stress group. This grouping was done according to the body weight of the rats. In each group, five rats were raised in a common animal room with a temperature of 22°C±2°C and a relative humidity of 30% to 40%. The rats were given conventional feed and free access to food and water. All animal experiments were carried out in accordance to the guidelines of China legislations on the ethical use and care of laboratory animals. All efforts were made to minimize animal suffering and the number of animals needed to produce reliable data.

### Chronic Immobilization stress procedure

The chronic immobilization method was applied as a chronic stress model. The rats were bound to a special binding rack composed of a type T binding base (20 cm×10 cm×5 cm) with a binding platform (length of 22 cm and maximum width of 6.6 cm). Grooves suitable for placing the limbs and two adjustable soft belts for fixing the chest and abdomen of the rats are located on both sides of the platform. The rats were placed into the binding rack for 3 h daily. The binding time points were random. In the binding process, the rats were in the same environment without food and water during 3 h immobilization stress, for 7 days (7-day stress group) or 21 days (21-dayl stress group). In the control group, the rats were not exposed to stress, with free access to food and water [12].

### Sampling

At forenoon of the second day after completion of the experiment for 7 or 21 days, 2% sodium pentobarbital was injected into the abdominal cavity of rats for deep anesthesia (40 mg/kg). The rats were decapitated and the hippocampus was dissected, on ice in Super-clean Bench, placed into liquid nitrogen, and transferred into a −80°C low-temperature refrigerator for storage and use.

### Reagents and instruments

Trizol, PrimeScriptTM RT Reagent kit, and SYBR ExscriptTM RT-PCR kit were provided by TaKaRa Company (Japan). Rat expression profile chip, hybridization kit, and chip tester, including a hybridization oven, a chip washing system, a chip scanner, and all other reagents were purchased from Illumina Company (USA).

### RNA extraction and mass spectrometry-based detection of hippocampus total RNA

Bilateral hippocampal tissue of nine rats were randomly selected using a random number table. Hippocampal total RNA was extracted using Trizol kit according to the manufacturer's instructions. An ultraviolet spectrophotometer was used to determine the purity and concentration of total RNA, and formaldehyde-denaturing agarose gel electrophoresis was performed to observe the integrity. The A_260_/A_280_ ratio values of all samples were between 1.96 and 2.02, and electrophoresis showed distinct 28 S and 18 S bands. The 28 S-to-18 S ratio was close to 2:1, which conforms to the requirements for the experiment. Total RNA was stored at −80°C for use.

### Analysis of the differential gene expression in the hippocampus

Three pooled RNA samples were obtained in each group, giving a total of nine total RNA specimens. For each specimen total RNA was taken. The Illumina chip experiment process was performed as follows: After conversion of the RNA to cDNA using RT-PCR and second strand cDNA synthesis, in vitro transcription was performed to generate and purificate cRNA, then detection of the purity and concentration of cRNA specimens using an ultraviolet spectrophotometer. Subsequently, cRNA was taken from each sample and hybridized with Rat Ref-12 BeadChip. Three arrays were used for each experimental group, and three biological replicates were performed. Illumina BeadChip Reader was used to read hybridized signals and acquire images, and Illumina BeadStudio Application was adopted to carry out data analysis and output results.

Differences between the experimental and control groups were considered significant at *P*<0.05. According to the reference literature [13], expression difference between two specimens was considered significant if the ratio of mean fluorescence intensity value of the experimental group gene/mean fluorescence intensity value of the control group gene (namely signal ratio value) was ≤0.67 or ≥1.5.

### Verification of partial differentially expressed genes of the hippocampus through real-time qPCR

Real-time qPCR was adopted to verify the up-regulation of *Gabra1* and *Fadd* gene expressions and the down-regulation of *Crhr2* and *Cdk6* gene expression. All selected RNA specimens were same as the ones from those used in the chip experiment. Primers were synthesized by Invitrogen Company. Real-time qPCR assay was conducted using SYBR Exscript^TM^ RT-PCR kit according to the manufacturer's instructions. Cycle threshold (Ct) was detected by monitoring changes in the fluorescence intensity signals using a GeneAmp 5700 fluorescent quantitative PCR instrument. The amounts of PCR reaction system, cDNA template sample, upstream primer, downstream primer, and SYBR Green I were 10, 1, 1, 1, and 0.5 µl, respectively. The reaction conditions were set as follows: denaturation for 2 min at 95°C; 30 cycles of 94°C for 10 s, 62°C for 10 s, and 72°C for 20 s; reading plate; and solubility curve at 55°C to 95°C. The reaction was terminated, and the temperature was reduced to 4°C. For mRNA of various genes, 2^−△△Ct^ was used to calculate its relative expression amount [14]. Data were expressed as mean ± standard deviation (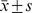); *P*<0.05 was considered to indicate statistically significant differences.

### Bioinformatic analysis of the differential gene expression profile of the hippocampus

1. The bioinformatics analysis tool, the database for annotation, visualization and integrated discovery (DAVID) and gene ontology (GO) [15] database were used to classify the bioprocess, molecular function, and cellular components of differentially expressed genes. A Fisher exact probability test was used.
2. According to bioinformatics classification and analysis of Kyoto encyclopedia of genes and genomes (KEGG, www.genome.jp/kegg/pathway.html) and Biocarta (www.biocarta.com) database on differential gene-involved signal pathways, all pathway information was provided by MSigDB database (www.broadinstitute.org/gsea/msigdb). Differential gene-involved significant signal pathways were determined according to the hypergeometric distribution.
3. Establishment of differentially expressed transcription regulation network. Comparative genomics and promoter region transcription factor binding site (TFBS) detection approaches were combined to establish a differential gene transcription regulation network. High-quality promoter region sequence was vital for predicting transcriptional regulatory events. Transcript factor (TF) mostly specifically bind the promoter region controlling transcription the corresponding target gene. For the establishment of transcription regulation networks, we first collected the promoter sequences. Data of rat promoter region sequences were obtained from UCSC (http://genome.ucsc.edu/) genome database. We focused on sequences from the 3000 bp upstream of the transcription start site (TSS) to 500 bp downstream and used them for TFBS motif discovery. UCSC provided 5000 bp to 1000 bp upstream sequences and the corresponding TSS as well as 500 bp downstream sequences were obtained using Rat4 genome sequence, resulting in total of 12,654 promoter sequences (−3000 bp to 500 bp). According to accession code number of differential genes of the 7-day and 21-day stress groups obtained through comparison with the normal group, we screened out the promoter sequences of these differentially expressed genes for prediction of the TFBS and their role in the regulation network. For motifs with lengths of 8, 10, 12, and 14 bp, we respectively recognized by TFBS. The main principle of the recognition algorithm was to take a promoter sequences of differentially expressed gene as positive data, herein referred to as Positive (Pos), and take non-differentially expressed genes (*P*-value was close to 1) with the same amount as the positive dataset randomly screened from genome as negative data, herein referred to as Negative (Neg). In the combination set of Pos and Neg, the basic group component was f. The maximum value complying with the objective function was recognized using the recurrence algorithm; that is, the optimal short motif was obtained. Among them, the optimization criterion was to obtain the optimum motif according to the maximum likelihood score ratio sorting.

For each group (differential gene-involved) of regulation events, the first 20 optimal motifs were selected. To reduce the occurrence of false positive results, highly reliable regulation TF and target gene were screened out. We carried out evolutionary conservative analysis of the motifs obtained by calculation and prediction on promoter sequences to screen more possible TF regulation regions. In addition, we conducted conservative analysis of the whole genome of eight vertebrate animals (mouse, human, dog, cow, opossum, chicken, frog, and zebrafish) in the last version of UCSC. The whole genome conservative analysis was completed using the phastCons software algorithm based on Two-State Hidden Markov Model (HMM) [16]. One state in the model was used for the conservative area, and the other was used for the non-conservative area. PhastCon score was calculated and obtained according to the ratio of average substitution rates of conservative area to non-conservative area. PhastCons used the state transition diagram based on phylo-HMM, including the state used for the conservative and non-conservative areas.

For each promoter region and binding site areas obtained using the motif probe algorithm (width  = 8 bp to 14 bp), we obtained the corresponding conservation of the respective motif (PhastCons value). Subsequently, we carried out a position weight matrix (PWM) analysis of the motifs obtained by prediction [17], [18] with TFBS binding sites in the latest TF databases TRANSFAC and JASPAR using the Motif Compare algorithm. The PWM similarity *P*-value obtained from the comparison should not exceed 10E-4 (or 10^−4^). At the same time, the corresponding TF binding motif sequence involved in the regulation was located in the conservative sequence and mapped onto the considered motif position via sequencing. As a result, the PhastCon Score of the corresponding TF binding motif sequence was ≥0.8 (1 represents the most conservative, whereas 0 represents the least conservative). For comparison of result from each group, the first five optimal TFs were output. Therefore, the TFs potentially regulating the differentially expressed genes of the 7-day and 21-day stress groups were obtained. Finally, according to the relationship between the TF and the target gene, differential gene-involved cis-transcription regulation networks of the 7-day and 21-day stress groups were established. The core genes in the network structure were calculated using the PageRank [19] and Cytoscape [20] (http://www.cytoscape.org/) software was used to conduct visualization of the regulation network.

## Results

### Gene chip detection

Compared with the control group, 602 differential genes in the 7-day stress group were identified, of which 387 were up-regulated and 215 were down-regulated. A total of 566 differential genes in the 21-day stress group were identified, of which 365 were up-regulated and 201 were down-regulated.

### GO function analysis

Significant bioprocesses of the differentially expressed genes of the hippocampus on the aspects of development, reproduction, biological regulation, bioadhesion, multicellular organism, cell process, localization, and establishment of localization, response to stress, immune system process, and so on (Table 1).

**Table 1:** Classification of the up-regulated and down-regulated genes involved in the significant bioprocesses of the 7-day and 21-day stress groups in comparison with the control group.

Term	Genes	Count	*P* Value
Up-regulated genes in 7-day stress group			
developmental process	2606	59	1.17E–06
biological regulation	5414	90	2.06E–04
multicellular organismal process	4351	76	2.60E–04
reproduction	678	16	0.020419
biological adhesion	463	12	0.028458
cellular process	7619	106	0.03951
response to stimulus	3867	59	0.045187
Down-regulated genes in 7-day stress group			
immune system process	715	13	5.26E–04
localization	2500	27	5.79E–04
establishment of localization	2145	20	0.022696
developmental process	2606	23	0.022955
Up-regulated genes in 21-day stress group			
biological regulation	5414	68	0.029596
Down-regulated genes in 21-day stress group			
immune system process	715	15	2.25E–05
developmental process	2606	25	0.003702
biological regulation	5414	41	0.005967
multicellular organismal process	4351	34	0.011826

### Signal pathway result of differential genes

**Figure 1:**
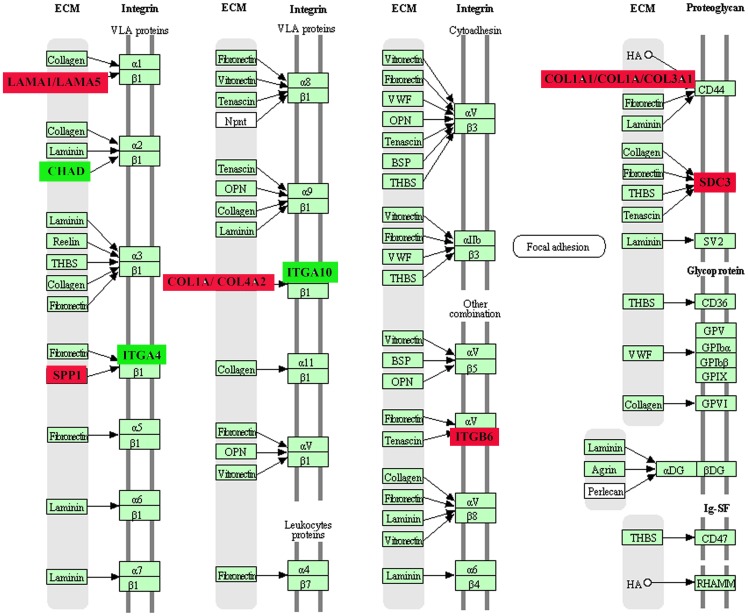
Extracellular matrix interaction signaling pathway. The most significant activate extracellular matrix receptor interaction path diagram of the 7-day stress group compared with the normal group. The red label for the channel of up-regulate genes: ITGB6, COL4A2, SPP1, LAMA1, LAMA5, COL1A1, COL1A, COL3A1, SDC3. The green label for the channel of down-regulate genes: ITGA10, ITGA4, CHAD.

**Figure 2:**
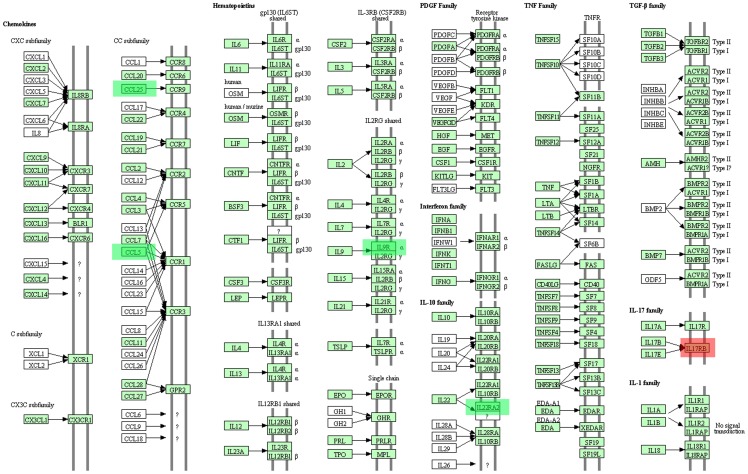
Cytokine-cytokine receptor interaction signaling pathway. The most significant inhibition of cell factor – cell factor receptor interaction path diagram of the 21- day stress group compared with the normal group. The green label for the channel of down-regulate genes: IL22RA2, IL9R, CCL5, CCL25. The red label for the path of the up-regulate gene: IL17RB.

Compared with the control group, the 7-day stress group had 11 differential gene-involved significant pathways. Among them, the extracellular matrix (ECM) receptor interaction pathway was significantly activated, with nine up-regulated genes and three down-regulated genes involved. The up-regulated genes were *Itgb6* (integrin, beta 6), *Col4a2* (collagen, type IV, alpha 2), *Spp1* (secreted phosphoprotein 1), *Lama1* (laminin, alpha 1), *Lama5* (laminin, alpha 5), *Col1a1* (collagen, type I, alpha 1), *Col1a* (collagen, type I, alpha), *Col3a1* (collagen, type III, alpha 1), and *Sdc3* (syndecan 3). The down-regulated genes were *Itga10* (integrin, alpha 10), *Itga4* (integrin, alpha 4), and *Chad* (chondroadherin) (Figure 1). The 21-day stress group had four differential gene-involved significant pathways in comparison with the control group. Among them, the cytokine-cytokine receptor interaction pathway was significantly affected. In this pathway, the up-regulated gene, *Il17rb* (interleukin 17 receptor B) and down-regulated genes *Il22ra2* (interleukin 22 receptor, alpha 2), *Il9r* (interleukin 9 receptor), *Ccl5*, and *Ccl25* were involved (Figure 2). Table 2 lists all the significant signal pathways indicated by differential gene (pathways with HAS prefix were from KEGG, and the other pathways without unified prefix were from Biocarta), differential gene number in the pathways, *P*-value and gene number of this pathway in the database.

**Table 2:** Analysis of the differential gene pathways of the 7-day and 21-day stress groups in comparison with the control group.

Pathways name	Genes in geneset (K)	Genes in overlap (k)	k/K	*P* value
7-DAY STESS GROUP				
ECM_RECEPTOR_INTERACTION	87	12	0.1379	7.36E–04
HSA04020- CALCIUM	174	2	0.0115	5.70E–03
HSA04810-REGULATION_OF_ACTIN_CYTOSKELETON	212	4	0.0189	1.23E–02
CALCIUM-REGULATION-IN-CARDIAC-CELLS	143	2	0.014	1.91E–02
GPCRDB-CLASS-A -RHODOPSIN- LIKE	185	4	0.0216	2.86E–02
HSA04010- MAPK	257	7	0.0272	2.98E–02
HSA04510–FOCAL- ADHESION	200	15	0.075	3.02E–02
HSA04360 –AXON- GUIDANCE	128	2	0.0156	3.30E–02
RIBOSOMAL- PROTEINS	123	2	0.0163	3.94E–02
HSA04310 -WNT	149	3	0.0201	3.94E–02
HSA01030-GLYCAN-STRUCTURES BIOSYNTHESIS	117	2	0.0171	4.84E–02
TERTPATHWAY	8	2	0.25	5.00E–02
21-DAY STRESS GROUP				
HSA04060-CYTOKINE_CYTOKINE_RECEPTOR_INTERACTION	257	5	0.0195	1.98E–02
HSA04810_REGULATION_OF_ACTIN_CYTOSKELETON	212	4	0.0189	2.95E–02
ACE_INHIBITOR	8	2	0.25	3.92E–02
SIG_PIP3_SIGNALING_IN_B_ LYMPHOCYTES	36	4	0.1111	4.82E–02

### Establishment of differential gene transcription regulation networks

**Figure 3:**
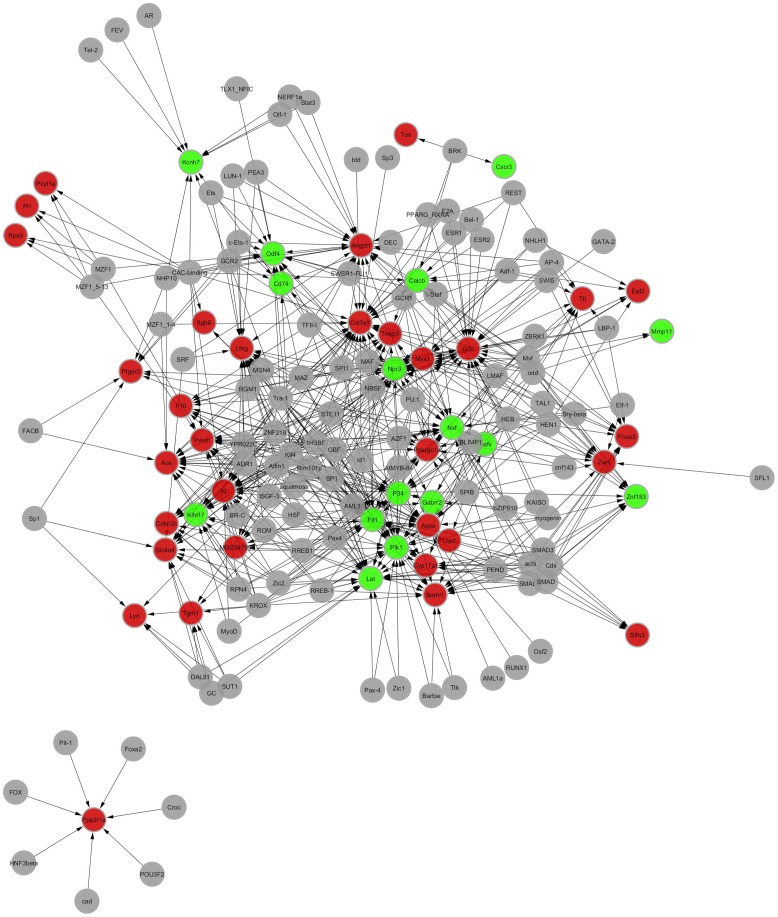
Visual diagram of the differential gene transcription regulation network of the 7-day stress group in comparison with the control group. The red represents up-regulate genes, the green represents down-regulate genes, the grey represents transcription factor. The arrow indicates the direction of TF target genes (TFT) regulated by transcription factor (TF).

**Figure 4:**
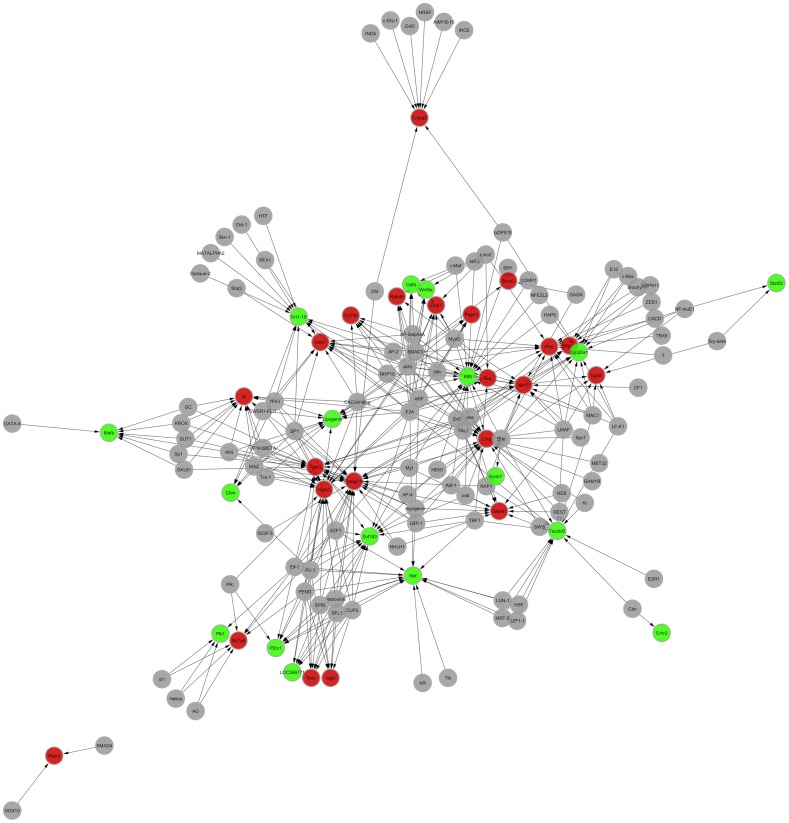
Visual diagram of the differential gene transcription regulation network of the 21-day stress group in comparison with the control group. The red represents up-regulate genes, the green represents down-regulate genes, the grey represents transcription factor. The arrow indicates the direction of TF target genes (TFT) regulated by transcription factor (TF).

The number of differential genes and TFs involved in the establishment of the transcription regulation network were respectively predicted by conducting promoter sequence analysis, TFBS prediction (Motif Discovery) for differential genes, and conservative analysis of genome cross species for the promoter and binding sites. Among the 602 differential genes in the 7-day stress group, 48 were involved in the establishment of transcription regulation network, of which 32 were up-regulated and 16 were down-regulated. A total of 112 TFs were predicted. In the transcription regulation network, 160 nodes (including TF and target gene) and 640 pairs of TF →TFT (TF target gene) potential relationships were involved. Among the 566 differential genes in the 21-day stress group, 39 were involved in the establishment of transcription regulation network, of which 22 were up-regulated and 17 were down-regulated. A total of 102 TFs were predicted. In the transcription regulation network, 141 nodes (including TF and target gene) and 384 pairs of TF→TFT potential relationships were involved. The PageRank values of the first 10 core genes in the network structure and their common TFs (Table 3). Regulation network structure diagrams are shown (Figure 3 and Figure 4). Red and green represent up-regulated and down-regulated genes, respectively, and gray represents TF. For regulation direction, TF to TFT was expressed by an arrow.

**Table 3:** PageRank values of the first 10 core genes in the network structure diagram and their common TFs.

Gene	Transcription factor	PageRank
7-day stress group		
Col3a1	ESR2, Myf, MAF, ESR1, Adf-1, SP1, PPARG_RXRA, BLIMP1, Alfin1, GCR1, SPIB, MAZ, TFII-I, Klf4, Bel-1, odd, MSN4, UF1H3BETA, Sp3, MZF1_1-4, EWSR1-FLI1, E2A, ADR1, Tra-1, RGM1, ISGF-3, id1, LMAF, ZNF219, TAL1, CAC-binding, AtMYB-84, btd, YPR022C.	0.045486
Ppp2r1a	cad, Pit-1, FOX, Foxa2, POU3F2, HNF3beta, Croc.	0.040471
Aspa	RREB-1, Alfin1, TAL1, HEN1, HEB, AML1a, RUNX1, squamosa, SPIB, Osf2, myogenin, BR-C, STE11, Klf4, Tra-1, ROM, Myf, HSF, odd, UF1H3BETA, SP1, AZF1, id1, Rim101p, AML1.	0.038828
G7c	TAL1, SWI5, myogenin, HEB, UF1H3BETA, AP-4, NRSF, REST, bZIP910, ESR2, Myf, MAF, HEN1, KAISO, LMAF, SPI1, SP1, AtMYB-84, ESR1, odd, ZNF219, BLIMP1, GATA-2, Bel-1, Klf4, PPARG_RXRA, MAZ, E2A, LBP-1.	0.037997
Nxf	Alfin1, MAF, AML1, GBF, id1, STE11, AZF1, UF1H3BETA, Pax4, PU.1, Klf4, SMAD, achi, GCR1, Cdx, ZBRK1, SMAD3, EWSR1-FLI1, PEND, Tra-1, TFII-I, Staf, Sry-beta, DEC, SMAD4, RREB1, znf143, Elf-1.	0.030808
Lfng	MAZ, Rim101p, BR-C, SP1, Tra-1, Klf4, EWSR1-FLI1, GCR2, ROM, Ets, SRF, STE11, UF1H3BETA, AZF1, HSF, Alfin1, RGM1, c-Ets-1, MSN4, YPR022C, ZNF219, PEA3, PU.1, SPI1, LUN-1, squamosa, ADR1.	0.030572
Angpt1	SRF, NRSF, TFII-I, LUN-1, odd, REST, SPI1, SWI5, Tra-1, NERF1a, NHP10, EWSR1-FLI1, Ets, c-Ets-1, HEB, Stat3, PEA3Olf-1, PU.1, Myf, GCR2, AP-4, LMAF, CAC-binding.	0.030465
Kcnh7	Olf-1, Tra-1, NERF1a, Tel-2, Stat3, TFII-I, CAC-binding, AR, NHP10, FEV.	0.029648
Odf4	Ets, TLX1_NFIC, GCR2, TFII-I, PU.1, LUN-1, PEA3, Tra-1, c-Ets-1, SPI1, EWSR1-FLI1, GCR1, Klf4, MAZ, Staf, MAF, MZF1, MZF1_5-13, DEC, CAC-binding.	0.0273
Lat	SP1, ADR1, RPN4, GBF, PEND, Pax-4, SUT1, AtMYB-84, PU.1, bZIP910, Tra-1, SMAD, SMAD3, ZNF219, YPR022C, KROX, RREB1, Zic2, Cdx, GC, LMAF, SMAD4, RREB-1, achi, Klf4, DAL81, SPIB, Alfin1, Zic1, UF1H3BETA, Pax4.	0.026438
21-day stress group		
Klf5	AP-2, v-Maf, LMAF, SRY, odd, Myf, AP-2alphaA, HAP5, COMP1, HEN1, NFE2L2, AZF1, NHP10, CAC-binding, MAC1, bZIP910, Dde, Nrf-2, Adf-1.	0.052951
Lfng	SWI5, E2A, Adf-1, myogenin, AP-2alphaA, LMAF, GAMYB, SMAD3, Myf, Spz1, AP-2, ARF, LBP-1, MET32, hth, achi, REST, odd, HEB, HEN1, AP-4, Kr, MyoD, MyoD, CAC-binding, NHP10.	0.049318
Cryba2	Dfd, bZIP910, NRSF, INO2, AtMYB-15, c-Ets-1, INO4, Osf2.	0.043825
Pter	Achi, Dde, Lmo2, ZEB1, sna, TBX5, E2A, Brachyury, MyoD, T, LMAF, SMAD3, CACD, MAC1, LF-A1.	0.0403
Ppargc1a	TAL1, SP1, MAZ, AP-4, RAP1, Tra-1, TFII-I, SPIB, ISGF-3, CUP2, Retroviral, PEND, myogenin, PU.1, AZF1, EWSR1-FLI1, UF1H3BETA, Klf4, LBP-1, NHLH1, Elf-1, Dfd, E47, sna, SFL1, CAC-binding, Myf, Dde.	0.039645
Krt1-19	Achi, TFII-I, EWSR1-FLI1, SP1, E2A, ARF, Skn-1, HTF, SMAD3, Oct-1, Opaque-2, MATALPHA2	0.036807
Tacstd2	Kr, Dde, E2A, MAC1, LMAF, HSF, TBF1, MEF-2, ESR1, SZF1-1, RAP1, Cdx, LUN-1, LF-A1.	0.033357
Nxf	ARF, MEF-2, KR, PEND, E2A, Retroviral, Ttk, SPIB, SFL1, TBF1, SZF1-1, LUN-1, CUP2, Elf-1, HSF, PU.1, AZF1.	0.031067
Tgm1	NHLH1, Sp1, AP-4, E47, myogenin, GC, AZF1, PEND, Klf4, sna, KROX, TAL1, SUT1, Myf, DAL81, LBP-1, Elf-1.	0.025603
Cyp26a1	LMAF, bZIP910, bZIP911, c-Myc, sna, MAC1, TAL1, E47, LF-A1, NF-muE1, E12, Dde, HAP5.	0.025388

### Real-time qPCR

The results of real-time qPCR assay coincided with the changes in differential gene identified by gene chip (Table 4).

**Table 4:** Relative quantitative analyses of the *Gabra1*, *Crhr2*, *Fadd*, and *Cdk6* genes expression in the rat hippocampal tissues of three groups.

Group	n	GABRA1		CRHR2		FADD		CDK6	
		2^−△△Ct^	Ratio	2^−△△Ct^	Ratio	2^−△△Ct^	Ratio	2^−△△Ct^	Ratio
Control	9	1.11±0.61	1.00	1.03±0.24	1.00	1.08±0.46	1.00	1.07±0.44	1.00
7-day	9	1.41±0.43	1.31	0.49±0.11^**^	0.52	1.89±0.52^**^	1.63	0.76±0.26	0.82
21-day	9	1.80±0.38^*^	1.55	0.43±0.05^**^	0.49	2.31±0.12^**^	1.96	0.44±0.13^**^	0.57

Compared with the normal group: ^*^ *P*<0.05, ^**^ *P*<0.01.

## Discussion

We used gene chip and bioinformatics technology to study the gene expression profile of the hippocampus of rats exposed to chronic immobilization stress for 7 or 21 days. Real-time qPCR was adopted to verify the up-regulation of *Gabra1* and *Fadd* gene expressions and the down-regulation of *Crhr2* and *Cdk6* gene expressions on the hippocampal tissues of various rat groups. These results coincided with the results of the gene chip assay, validating the gene chip assay results. Previous studies showed that the down-regulation of *Crhr2* gene expression could enhance the activity of rat HPA axis, blood plasma adrenocorticotropic hormone, and corticosterone levels after the stress reached the peak more rapidly than those of the control group [21]. CRHR2 could maintain and regulate the effect of the HPA axis [22] and could participate in the recovery regulation of HPA axis response [23]. The level of blood plasma corticosterone in the experimental rats with *Crhr2* deficiency was still high 90 min after stress. The *Crhr2* gene expression of the hippocampal tissues in the 7-day and 21-day stressed rats was significantly down-regulated. This result suggests that the daily exposure to stress for 3 h causes functional disorder in the HPA axis of the stressed rats.

In line with previous work [24]–[28], we observed that exposure to stress for 7 or 21 days significantly inhibited the function of the “immune system process” in rat hippocampi, but the inhibiting effect of the 21-day stress was more significant than that of the 7-day stress. Studies have shown that a mutual relationship exists between the hippocampus and the body's immune system. However, the results of different studies are inconsistent [29]–[31]. This discrepancy in results may be attributed to the duration and intensity of stress exposure, as well as to the single or multiple genes and proteins related to observed indicators. This study also showed that the regulating effect of the hippocampus on the immunologic function was extremely complex. Moreover, multiple genes jointly participated in the immunologic response process and inhibited the function of the immune system process.

For the 7-day stressed hippocampus, the down-regulated genes that significantly inhibited the immunologic response were complement component 5 (*C5*), myosin IF (*Myo1f* ), RT1 class II, locus Ba (*Rt1-ba* ), chemokine (C-C motif) ligand 5 (*Ccl5)*, CD74 antigen (*Cd74* ), RT1 class II, locus Da (*Rt1-da* ), telomerase RNA component (*Terc* ), chemokine (C-C motif) ligand 25 (*Ccl25)*, linker for activation of T cells (*Lat* ), CD34 antigen (*Cd34*), lymphocyte-specific protein tyrosine kinase (*Lck* ), zeta-chain (TCR) associated protein kinase 70 kDa (*Zap70)*, member RAS oncogene family (*Rab27a* ), and RT1 class I, CE7 (*Rt1-ce7* ). For the 21-day stressed hippocampi, the down-regulated genes that significantly inhibited the immunologic response were C-reactive protein, pentraxin-related (*Crp* ), signal transducer and activator of transcription 5B (*Stat5b* ), *C5*, cyclin-dependent kinase 6 (*Cdk6*), *Ccl5*, dopamine beta hydroxylase (*Dβh* ), *Terc*, RT1 class I, A3 (*Rt1-a3*), *Ccl25*, 2′–5′ oligoadenylate synthetase 1I (*Oas1l* ), CD34 antigen (*Cd34*), interferon regulatory factor 7 (*Irf7*), erythroid associated factor (*Eraf* ), and defensin beta 1 (*Defβ1* ).

We analyzed these differential genes of down-regulated expression and found that the main manifestations of the 7-day and 21-day stressed hippocampi-inhibiting function of the immune system process lay in the following aspects. First, both 7-day and 21-day stresses inhibited the complement-activating cascade reaction, hematopoietic progenitor cell formation, T progenitor cell thymus nesting, and increase in dendritic cell (DC) number and telomerase activity. Complement C5, CD34 antigen, chemotactic factors CCL5 and CCL25, and telomerase RNA component (TERC) were down-regulated during the 7-day and 21-day stress processes. Human telomerase RNA (hTR) and mouse telomerase RNA (mTR) have 65% sequence similarity [32]. Telomerase RNA acts as the main telomerase structure, whereas hTR is the telomerase RNA template that synthesizes telomere. During template region mutation or deficiency and alteration in transfected cell telomere sequences, cells undergo apoptosis or differentiation [33]. Telomerase RNA is a subunit necessary for telomerase activation. Hence, inhibition of telomerase activity can cause delay in G1 stage and growth retardation [34], [35]. Therefore, the down-regulation of *Terc* gene expression promotes growth retardation and immunocyte apoptosis, which further negatively regulates immunologic response.

Second, both 7-day and 21-day stresses affected the activation of CD4+ T and CD8+ T lymphocytes. The down-regulation of the expression of MHC II (Major Histocompatibility Complex) genes *Rt1-ba* and *Rt1-da* and MHC-II constant – chain CD74 molecule in the 7-day stress, as well as the down-regulation of the expression of MHC I genes *Rt1-ce7* and *Rt1-a3* in the 21-day stress, affects the activation of CD4+ T and CD8+ T cells. MHC II and I molecules are cell surface glycoproteins that mediate antigen presentation, which is the function of the immune system. MHC II molecules present exogenous antigen peptide to CD4+ T cells [36], [37], whereas MHC I molecules present cytoplasmic antigen peptide to CD8+ T lymphocytes [38]. The 7-day and 21-day stresses may change the CD4+ T cell-to-CD8+ T cell ratio. Balance in the intracorporal CD4+ T/CD8+ T lymphocyte subpopulation is vital in maintaining the body's immune balance.

Third, the 21-day stress inhibited the immune system process more extensively than the 7-day stress. This finding was determined from the following manifestations. 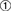 Immunologic response is more significant in retardation manifestations of immune physiological resistance such as the activation, proliferation, and development of T cell and signal transduction in the early stage of stress, as well as growth retardation of immunocytes in the later stage of stress. Linker expressions for the activation of T cells (LAT) ZAP70 [zeta-chain (TCR)-associated protein kinase 70 kDa] are down-regulated during the early stage of stress, and CDK6 expressions are down-regulated in the later stage of stress. ZAP70, a protein tyrosine kinase (PTK) associated with T lymphocyte receptor (TCR), plays an important role in the initiation, activation, and phosphorylation of signal transduction in multiple downstream target genes [39]. Moreover, the intracellular transduction of TCR activation signal depends on the transduction of activated ZAP-70 phosphorylation adapters LAT and SLP-76. LAT can be a signal platform for integrating and distributing signals from TCR [40], [41] and can affect the biochemical signals connecting TCR with activated T cells. The down-regulation of ZAP70 and LAT expressions retards the transduction of T cell signal to a certain extent, thereby inhibiting the activation capacity of T lymphocytes. The encoding product of the *Cdk6* gene is pivotal in cell cycle regulation, especially in the transition from the Gl stage to the S stage. *Cdk6* activity reduction or inhibition can prevent the cells in the G1 stage from entering the S stage, thus inhibiting cell proliferation, retarding cell growth, and causing cell senescence [42]. The down-regulation of CDK6 expression results in growth retardation and immunocyte hypofunction, causing changes in the function of the hippocampus to regulate immunologic response. 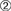 An immunosuppression cascade effect occurred during the 7-day and 21-day stress processes. The expression of lymphocyte-specific protein tyrosine kinase (Lck) is down-regulated in the 7-day stress process, whereas that of *Stat5b* is down-regulated at the end of the 21-day stress. Lck is a protein tyrosine kinase from the Src family. It is mainly present in T lymphocytes because it participates in the signal transduction of T cell development, differentiation, and activation [43]. STAT5B is necessary for the behavior of eosinophilic granulocyte and T cell gathering towards tissues induced by antigen [44], and is involved in the differentiation of B and T lymphocytes. The amount of B lymphocytes in the peripheral blood of *Stat5b*-deficient mice is obviously reduced [45]. Although the current research lacks explanation on the occurrence of Lck and STAT5B interaction in cells, STAT5B is confirmed to be the downstream signal molecule that greatly affects the cell bioprocess induced by Lck activation. 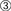 The down-regulation of defensin beta 1 (DEFβ1) expression caused by the 21-day stress affects DC maturation. DEFβ1 can chemotactically induce young DCs and memory T cells to gather into inflammation sites, mediating young DC maturation, further activating T cells, and triggering powerful intracorporeal immunologic response. Therefore, DEFβ1 plays an important role in both inherent and acquired immunity [46], [47]. 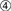 The 21-day stress down-regulates the function of interferon (IFN). The 7- and 21-day stresses up-regulated and down-regulated the expression of interferon regulation factor 7 (IRF7), respectively. The proinflammatory gene C-reactive protein (CRP) is also down-regulated after the 21-day stress. IFN has two types: type I (IFN-α and IFN-β) and type II (IFN-γ). Aside from resisting viral and bacterial infections, type I IFN (IFN-a/B) also plays a role in the regulation of adaption, inherent immunologic reaction, cell growth, and cell differentiation [48]. CRP is the main acute-phase protein during infection, inflammation, and tissue damage, serving as a natural immune molecule and proinflammatory medium that actively participates in its own inflammation process [49]. CRP has many characteristics similar to immunoglobulin IgG. When combined with a specific ligand, it can activate the classical complement pathway and the mononuclear macrophage to promote inflammatory reaction expansion through Fcr receptor (the receptor that binds different immunoglobulins such as IgA, IgE, IgM, and IgG) and complement receptors [50], [51]. Previous studies showed that the down-regulation of acute proinflammatory gene expression is involved in maintaining immunosuppression and is a warning signal of death risk [52].

During the 7-day and 21-day stress processes, inhibition of the immunologic response of the hippocampus is caused by the combined action of polygenic, multilevel, and multi-signal pathways. Approximately 70% and 25% of T cells belong to CD4+ T accessory cells (Th) and CD8^+^T suppressor cells, respectively. Previous studies mainly focused on CD4+ T accessory cells (Th). Traditionally, T cells are divided into Th1 and Th2 subpopulations according to the generated cytokine profile and its biological function [53]. Th1 cells secrete cytokines that mediate cellular immunologic response, whereas Th2 cells secrete cytokines that mediate the immunologic response of bodily fluids [54], [55]. Moreover, activated signal transducer and activator of transcription 4 (STAT4) promotes Thl cell differentiation, whereas activated STAT6 promotes Th2 cell differentiation [56]. In recent years, the concept that CD4+ T cells are divided into Th1 and Th2 subpopulations has been constantly updated with the continuous improvement in technology. CD4+T can differentiate into Th1, Th2, Treg (a class of regulatory T cells), Th17, Th9, and Th22 subpopulations with different immunologic functions *in vivo*. Th17 mainly secretes IL-17 [57], whereas Th9 cell mainly secretes IL-9 and IL-10 [58]. Furthermore, IL-22 is the main effector for Th22 cell-realizing immunoregulation [59], [60]. During stimulus or alien antigen attack, the function of either Th1 or Th2 subpopulation is increased, whereas that of other subpopulations is reduced, causing Th1-Th2 disequilibrium or the so-called “immunologic drift” phenomenon [61]. In that way, which immunologic drift changes are hippocampus Th cells of chronic Immobilization stress rats in this study subject to?

In the above analysis, we discussed the down-regulation of CCL5 or monocyte chemoattractant protein-1 (MCP-1) expression during the 7-day and 21-day stress processes. Chiu et al. revealed that the expression of MCP-1 is up-regulated in the Th1-dominant response state (Th1 cell-mediated cellular immunologic response) but down-regulated in the Th2-dominant response state [62]. Therefore, the 7-day and 21-day stresses down-regulate hippocampal Th1 cell-mediated cellular immunologic response and up-regulate Th2 cell-mediated immunologic response. In addition, IL-13RA2 expression is up-regulated and interleukin 22 receptor, alpha 2 (IL-22RA2) expression is down-regulated in the 7-day stress. IL-9R and IL-22RA2 expressions are down-regulated and IL-17RB expression is up-regulated in the 21-day stress. The up-regulation or down-regulation of the expressions of IL-13RA2, IL-22RA2, IL-9R, and IL-17RB receptors indicates that the corresponding IL-9, IL-22, IL-13, and IL-17 expressions in the hippocampal tissue of the stressed rats are either inhibited or activated. Therefore, the above analysis of Th cell classification clearly implies that the disequilibrium of hippocampal Th cells Th1/ Th2 occurs in the 7-day and 21-day stress processes and that Th1 and Th2 chemotactically drift towards Th2 to inhibit the immunologic function of Th22 cells. The 21-day stress continuously inhibits the immunologic function of Th9 cells but enhances the immunologic function Th17 cells. Furthermore, the expression of STAT6 is up-regulated in the 7-day stress, whereas that of STAT4 is down-regulated in the 21-day stress. Therefore, Th cells are speculated to drift towards Th2 more significantly in the 7-day stress than in the 21-day stress, whereas the 21-day stress inhibits Th1 cell more significantly than the 7-day stress. In addition, during the 21-day stress, IL-22RA2 and IL-17RB show opposite expressions. Moreover, IL-22RA2 expression is down-regulated more significantly in the 7-day stress (ratio: 0.42) than in the 21-day stress (ratio: 0.52). Therefore, this study speculates that IL-22RA2 mainly resulted from Th22 cell expression rather than from Th1 and Th17 cell expressions.

We have continuously clarified the immunologic drift phenomenon of hippocampus Th cells in stressed rats. The most fundamental reason for the immunologic drift has yet to be determined. Previous studies suggest that the effects of stress on the body are mainly caused by increased endogenous GC, due to stress reaction or exogenous GC for clinical treatment. The changes in the intracorporeal GC level that cause abnormal cytokine expression, thereby resulting in cytokine balance network disorder and various diseases [63]. GC can make the Th1 cell-mediated immunologic response [64], [65] drift towards Th2 [66], and its induced Th1/Th2 expression status primarily causes low clinical immunity. A previous study reported that the pharmacological concentration of GC could inhibit chemotactic function [67]. GC strongly disturbs DC differentiation and maturation [68], causing decrease in DC antigen-presenting capacity [69] and inhibiting the capacity of DC-activating T cells [68]. As an anti-inflammatory agent, GCs can down-regulate the expressions of some proinflammatory factors, such as IL-1β, TNF-β, interferon–α (IFN-α), and IFN-β, among others [70]. Therefore, we have adequate evidence to speculate that the increase in the amount of GCs during the 7-day and 21-day stresses also caused the inhibition of the immunologic function of the hippocampus.

The effect of the increase in GC amount on the hippocampus was determined through gene transcription regulation network analysis. For the 7-day regulation network, the first 10 core genes with the largest Page Rank weight are *Col3a1*, *Ppp2r1a*, *Aspa*, *G7c*, *Nxf*, *Lfng*, *Angpt1*, *Kcnh7*, *Odf4*, and *Lat*. GC and its receptor I (mineralocorticoid receptor, MR) and receptor II (GR) are involved in the transcriptional regulation of the *Col3a1*, *Nxf*, *Odf4*, *Lat*, *Lfng*, and *Angpt1* genes. These findings suggest that the stressed rats presented enhanced neuroendocrine response during the early stage of chronic stress (7 days). The level of endogenous stress hormone GC is increased, the corresponding GR and MR levels in the hippocampus are increased, and the regulation capacity of the hippocampus to stress is enhanced. The 7-day stress significantly up-regulates the bioprocess function of “stress response”. The collagen synthesis ability of hippocampal tissues is the core event in the regulation network structure, and the up-regulation of *Col3a1* gene expression is at the most central position of the network. However, MR and GR are not shown in the 21-day regulation network. Only GC has a regulating effect on the network structure. Therefore, during the 21-day chronic immobilization stress, the level of GCs remains high because of the persistent existence of stressor and large cumulative intensity, where GR and MR show a depletion phenomenon. The hippocampus has the highest content of GRs in the central nervous system, and it is the high regulation center for HPA axis stress reaction. GC regulates the excitability of the HPA axis in the negative feedback form by combining hippocampus GR [71]. The reduction in the number of GRs in the hippocampus attenuates the negative feedback function of the hippocampus to the HPA axis and further exacerbates high corticosterone ketosis and forms a cycle, prolonging the exposure of the hippocampus to high-level cortisol. Various chronic stress animal models show that high-level GC stress causes lasting hippocampal neuron damage, possibly decreasing hippocampal volume [72]. Moreover, hippocampal neuron damage further reduces the hippocampal inhibition of the HPA axis. In the network structure, high-level GC causes hippocampal cell damage, and the Kruppel-like factor 5 (*Klf5*) gene with a down-regulating expression is at the most central position in the network. We assume that *Klf4* is involved in regulation network establishment. KLF4 and KLF5 are members of the Kruppel-like transcription factor family that are involved in the cycle, proliferation, differentiation, apoptosis, and growth of cells. Generally, KLF4 inhibits cell growth, whereas KLF5 stimulates cell proliferation [73]. In the network structure, the expressions of *Klf4*-regulating target gene homeodomain interacting protein kinase 3 (*Hipk3*), transglutaminase 1 (*Tgm1*), peroxisome proliferative activated receptor, gamma, co-activator 1 alpha (*Ppargc1a*), and androgen receptor (*Ar*) are up-regulated. Existing studies suggest that HIPK3 plays an important role in FAS-mediated apoptosis. In the current study, FADD expression is up-regulated in the 7-day and 21-day stress processes. The up-regulation of FADD expression in the 21-day stress process is more significant than that in the 7-day stress process and is closely related to the up-regulation of HIPK3 expression. FADD is the main signal transduction protein for Fas/FasL-mediated apoptosis. FADD, Fas, and FasL form a trimer. The interaction of HIPK3-FADD activates FADD and enhances the apoptosis signal transduction. Therefore, the 21-day stress breaks the balance in normal hippocampal cell growth and apoptosis, suppresses hippocampal cell growth, hastens apoptosis, and causes numerous hippocampal cell losses, thereby damaging the function of the hippocampus. The GO function analysis suggests that after the 21-day stress, hippocampal functions are damaged. Hence, the regulation capacity of the hippocampus to stress reaction is very limited. The mechanism of chronic immobilization stress that results in a great amount of hippocampal cell loss is analyzed through signal pathway analysis.

Signal pathway analysis shows that in the early stage of stress (7 days), the functions of multi-signal pathways are significantly activated. Among the 12 genes involved in the ECM receptor interaction pathway, 9 are up-regulated and 3 are down-regulated. This finding suggests that in the 7-day stress process, ECM synthesis and degradation of the hippocampal tissue are out of balance. ECM generation and degradation are increased and reduced, respectively, causing excessive deposition of ECM in the hippocampal tissue.

The up-regulated expressions of the genes on the pathways are involved in integrin and its ligand ECM proteins, such as collagen, laminin, osteopontin, and heparan sulfateproteoglycans SDC3 (Syndecan-3), and other components. Among them, collagen is the main component, which is involved in the activation of type I, III, and IV collagen, and down-regulated genes include integrin. Previous studies proved that activated integrin promotes axon growth [74] and intracorporeal neural regeneration [75]. Cells separated from adhesion matrix and lost intercellular linkage, which would cause anoikis [76]. Integrin could promote cell movement to avoid anoikis. Both the degradation of laminin and its disturbance to the relationship between cells and laminin can cause nerve cell apoptosis [77]. SDC3 is involved in CNS establishment [78], and SDC3 expression up-regulation possibly plays a role in repairing damaged hippocampus. Collagen, the main structural component of the ECM, is also essential in mitogen-stimulated cell cycle [79]. However, some reports showed that collagen could suppress cell proliferation [80]. In organ fibrosis, excessive deposition of fibrillar collagen, such as type I collagen, causes sclerosis of tissues and organs and finally leads to functional loss. The activation of type I collagen COL1A1 and COL1A of hippocampal tissues causes hippocampal sclerosis to a certain extent. Hippocampal sclerosis was firstly proposed by Falcomer et al [81]. The main pathological manifestations of hippocampal sclerosis lay in hippocampal atrophy and neuron loss, as well as colloid proliferation in some regions of the hippocampal structure. Thus, the activation of hippocampal ECM in the 7-day stress clearly promotes the self-repair of damaged hippocampal cells, promoting hippocampal sclerosis and causing partial neuron loss. The Th2 immunologic drift occurs in the 7-day stress process, and type Th2 cytokine is involved in the immunologic response of bodily fluids and inhibits acute and chronic inflammatory reactions [54]. These findings suggest that chronic inflammatory reactions occur in hippocampal tissues during the early stage of chronic Immobilization stress. Excessive ECM deposition is the compensatory reaction of hippocampal tissue repair process secondary to hippocampal inflammation, and damaged hippocampal cells promote ECM synthesis for self-repair.

As a persistent stressor during the 7-day and 21-day stress processes, increased amount of GCs causes the denaturation and death of hippocampal neurons, and the activation of the calcium pathway results in intracellular calcium overload and nerve cell necrosis [82]. The activation of the p38 MAPK pathway is involved in Fas-mediated apoptosis, and FADD activation and CDK6 inhibition suppress hippocampal cell growth, hasten apoptosis, and cause a great amount of hippocampal cell loss. Therefore, at the end of the 21-day stress process, the resistance of the hippocampus to stress and the activation of signal pathways are obviously reduced. The activation of the ECM receptor interaction pathway cannot present significant changes. The 21-day stress significantly inhibits the function of cytokine receptor interaction pathway. In the pathways, the expressions of CCL5, CCL25, IL-9R, and IL-22RA2 are down-regulated, whereas the expression of IL-17RB is up-regulated. Aside from their immunologic functions, cytokines play important roles in inflammation reaction. Inflammatory factor binds the corresponding receptor of the central nervous system and activates small colloid cells and vascular endothelial cells, triggering a series of inflammatory reactions [83]. In addition, the hippocampus is the gathering area of the cerebral inflammatory factor receptor expressions and is more sensitive to excessive or chronic inflammatory factor expressions [84]. IL-17RB up-regulate expressions in low level and inhibits the general activation of inflammatory factors. This finding may be attributed to the anti-inflammatory effect of GCs. Previous studies suggested that CCL5 and CCL25 are usually in a high-expression state during an autoimmune disorder. IL-9 [85], [86], IL-22 [87], and IL-17 [88] are proinflammatory factors that present high-level expressions in various chronic inflammation diseases. During the 21-day stress process, IL-17RB activation suggests that the hippocampal tissue is chronically inflamed. Moreover, inflammatory reactions in the central nervous system can cause a great amount of apoptosis. Non-scavenged apoptotic cells or apoptotic bodies possibly increase the existence of autoantigen peptides and cause autoimmune damage. Therefore, Th17/IL-17 is a possible target in treating chronic inflammation diseases and IL-17-associated autoimmune disorders [89].

In the 21-day stress process, hippocampal tissues continuously demonstrate chronic inflammatory reactions, hippocampal ECM are increased, and the calcium ion pathway and MAPK signal pathway remain activated. The suppression of hippocampal Th1 cytokine is more significant in the 21-day stress process than in the 7-day stress process. Type Th1 cytokine can promote the repair of normal tissue structure, and the growth of 21-day stressed hippocampal cells is also significantly inhibited. These factors cause the denaturation and loss of hippocampal neurons, eventually leading to hippocampal sclerosis, irreversible disorganization, and functional damage of hippocampal tissues. In recent years, neuroimaging technology has shown that chronic stress causes changes in the brain structure, including the reduction in hippocampal volume [90], which is related to the duration of stress exposure [91].

After investigating the gene expression profile of the hippocampi of 7-day and 21-day stressed rats, we suggest that the combined action of polygenic, multilevel, and multi-signal pathways inhibits the immunologic functions of the hippocampus. Moreover, the balance of hippocampal apoptosis and proliferation is disrupted. The immunologic functions of the Th1, Th2, and Th22 cells of the hippocampus are altered during 7-day stress process, whereas those of the Th1, Th2, Th22, Th9, and Th17 cells are changed during the 21-day stress process. The functions of the ECM receptor action signal pathway and cytokine-cytokine receptor interaction signal pathway are significantly changed, which is closely associated with the suppression of the immunologic functions of the hippocampus, as well as the balance disorder in hippocampal apoptosis and proliferation. The changes in the functions of the hippocampus are mainly attributed to the persistent chronic inflammatory reaction of hippocampal tissues. Long-term chronic stress is related to the body's autoimmune disorder, which can be ascribed to the increase in the amount of GCs. FADD, Col3a1, KLF5, and IL-17RB genes are important in the stress reaction. The current study not only verifies and improves the existing research results but also explains some contestable problems. Recommendations for future studies include verification and in-depth analysis of differential gene expression profile to provide a new direction for the gene therapy of chronic stress diseases.
